# Temporal Changes of Human Breast Milk Lipids of Chinese Mothers

**DOI:** 10.3390/nu8110715

**Published:** 2016-11-10

**Authors:** Francesca Giuffrida, Cristina Cruz-Hernandez, Emmanuelle Bertschy, Patric Fontannaz, Isabelle Masserey Elmelegy, Isabelle Tavazzi, Cynthia Marmet, Belén Sanchez-Bridge, Sagar K. Thakkar, Carlos Antonio De Castro, Gerard Vinyes-Pares, Yumei Zhang, Peiyu Wang

**Affiliations:** 1 Nestlé Research Center, Nestec Ltd., Vers-chez-les-Blanc, P.O. Box 44, 1000 Lausanne 26, Switzerland; cristina.cruz-hernandez@rdls.nestle.com (C.C.-H.); emmanuelle.bertschy@rdls.nestle.com (E.B.); patric.fontannaz@rdls.nestle.com (P.F.); isabelle.masserey-elmlegy@rdls.nestle.com (I.M.E.); isabelle.tavazzi@rdls.nestle.com (I.T.); cynthia.marmet@rdls.nestle.com (C.M.); belen.sanchez-bridge@rdls.nestle.com (B.S.-B.); sagar.thakkar@rdls.nestle.com (S.K.T.); carlosantonio.decastro@rdls.nestle.com (C.A.D.C.); 2 Nestlé Research Center Beijing, Building E-F, No. 5 Dijin Road, Haidian District, Beijing 100091, China; gerard.vinyespares@nestle.com; 3 Department of Nutrition and Food Hygiene, School of Public Health, Peking University Health Science Center, Beijing 100191, China; zhangyumei@bjmu.edu.cn; 4 Department of Social Medicine and Health Education, School of Public Health, Peking University Health Science Center, Beijing 100191, China; wpeiyu@bjmu.edu.cn

**Keywords:** FA, phospholipids, gangliosides, breast milk, chromatography

## Abstract

Fatty acids (FA), phospholipids (PL), and gangliosides (GD) play a central role in infant growth, immune and inflammatory responses. The aim of this study was to determine FA, PL, and GD compositional changes in human milk (HM) during lactation in a large group of Chinese lactating mothers (540 volunteers) residing in Beijing, Guangzhou, and Suzhou. HM samples were collected after full expression from one breast and while the baby was fed on the other breast. FA were assessed by direct methylation followed by gas chromatography (GC) analysis. PL and GD were extracted using chloroform and methanol. A methodology employing liquid chromatography coupled with an evaporative light scattering detector (ELSD) and with time of flight (TOF) mass spectrometry was used to quantify PL and GD classes in HM, respectively. Saturated FA (SFA), mono-unsaturated FA (MUFA), and PL content decreased during lactation, while polyunsaturated FA (PUFA) and GD content increased. Among different cities, over the lactation time, HM from Beijing showed the highest SFA content, HM from Guangzhou the highest MUFA content and HM from Suzhou the highest *n*-3PUFA content. The highest total PL and GD contents were observed in HM from Suzhou. In order to investigate the influence of the diet on maternal milk composition, a careful analyses of dietary habits of these population needs to be performed in the future.

## 1. Introduction

Human milk (HM) is considered the optimal form of nourishment for infants during the first six months of life [1] and among its macronutrients, the lipid fraction is crucial, representing approximately 50% of the energy supplied to the newborn infant [2]. Lipids (2%–5%) occur in milk in the form of fat globules mainly composed of triacylglycerols (TAG) (~98% of total lipids) surrounded by a structural membrane composed of phospholipids (PL) (0.8%), cholesterol (0.5%), enzymes, proteins, glycosphingolipids (e.g., gangliosides (GD)), and glycoproteins [3,4].

The majority of fatty acids (FA), approximately 98%, are esterified to a glycerol backbone to form TAG and about 0.2%–2% is found in molecules, such as cholesterol, PL, and GD. In HM, saturated FA (SFA) content ranges from 20% to 70% of total FA, mono-unsaturated FA (MUFA) from 23% to 55%, polyunsaturated FA (PUFA) from 6% to 36%, and long chain polyunsaturated FA (LCPUFA) from 0.3% to 8%. Among PUFA, linoleic (LA, 18:2*n*-6) and alpha linolenic acids (ALA, 18:3*n*-3) are essential because they are not synthesized in the human body and they are precursors of arachidonic (ARA, 20:4*n*-6) and docosahexaenoic (DHA, 22:6*n*-3) FA that are associated with normal brain development, especially in early life [5].

PL are mainly distributed into five classes: phosphatydylinositol (PtdIns), phosphatydylethanolamine (PtdEtn), phosphatydylserine (PtdSer), phosphatidylcholine (PtdCho), and sphingomyelin (CerPCho). Ptdlns, PtdEtn, PtdSer, and PtdCho consist of a glycerol esterified with FA in the *sn*-1 and *sn*-2 positions. A phosphate residue with different organic groups (inositol, serine, ethanolamine, or choline) is present in the *sn*-*3* position. CerPCho consists of a sphingoid base backbone to which an amide-linked long-chain FA can be attached, leading to the ceramides (*N*-acyl-sphingoid bases) [6]. In the case of CerPCho the primary hydroxyl group of the sphingoid base is linked to phosphorylcholine. Therefore, PL are a source of FA and choline, the precursors of the neurotransmitter acetylcholine, which acts by regulating the transduction signal and serves as a source of methyl groups in intermediate metabolism, being considered essential for optimum development of the brain [7,8].

GD are glycosphingolipids formed by a hydrophobic ceramide and a hydrophilic oligosaccharide chain. This chain may contain *N*-acetylneuraminic acid (sialic acid) or, less commonly, *N*-glycoloylneuraminic acid (Neu5Gc), where a glycol group is bound to the C5 amino group. It has been reported that sialic acid is involved in many biological and pathological phenomena, either recognizing or masking the recognition of several ligands, such as selectins or pathogens [9]. Recently, Gurnida et al. [10] concluded that nutritional supplementation with a milk lipid preparation rich in GD appears to have beneficial effects on cognitive development in healthy infants aged 0–6 months. 

Traditionally, lactation has been viewed in three stages: colostrum (day 1–5 postpartum), transitional milk (day 6–15 postpartum), and mature milk (after day 15 postpartum). It has been showed that FA, PL, and GD content in HM change during lactation stages [4,11,12,13,14,15,16,17,18,19] and factors, such as maternal diet. may influence HM short chain FA [20,21], PUFA composition [22,23,24], and gangliosides content [25].

The objective of this study was to determine, for the first time, the FA, PL, and GD content in HM of Chinese mothers, follow its temporal change along lactation, and evaluate if the geographical region within China would affect HM lipid composition. This study is part of the larger initiative: the Maternal Infant Nutrition Growth (MING) study [26].

## 2. Materials and Methods 

### 2.1. Subjects

This study was part of MING, a cross-sectional study designed to investigate the dietary and nutritional status of pregnant women, lactating mothers, and young children aged from birth up to three years living in urban areas of China [26]. In addition, the HM composition of Chinese lactating mothers was characterized. The study was conducted between October 2011 and February 2012. A multi-stage milk sampling from lactating mothers in three cities (Beijing, Suzhou, and Guangzhou) was performed for breast milk characterization. In each city, two hospitals with maternal and child care units were selected and, at each site, mothers at lactation period 0–240 days were randomly selected based on eligibility criteria. Subjects included in the period 0–5 days were recruited at the hospital, whereas the other subjects were requested by phone to join the study; if participation was dismissed a replacement was made. The response rate was 52%. Recruitment and milk, as well as baseline data collection, were done in separate days. A stratified milk sampling of 540 lactating mothers in six lactation periods of 0–4, 5–11, and 12–30 days, and 1–2, 2–4, and 4–8 months was obtained in the MING study. 

### 2.2. Inclusion and Exclusion Criteria

Eligibility criteria included women between 18–45 years of age giving birth to a single, healthy, full-term infant and exclusive breastfeeding at least until four months of age. Exclusion criteria included gestational diabetes, hypertension, cardiac diseases, acute communicable diseases, and postpartum depression. Lactating women who had nipple or lacteal gland diseases, who had been receiving hormonal therapy during the three months preceding recruitment, or who had insufficient skills to understand study questionnaires were also excluded.

### 2.3. Ethical and Legal Considerations

The study was conducted according to the guidelines in the Declaration of Helsinki. All of the procedures involving human subjects were approved by the Medical Ethics Research Board of Peking University (No. IRB00001052-11042). Written informed consent was obtained from all subjects participating in the study. The study was also registered in ClinicalTrials.gov with the number identifier NCT01971671.

### 2.4. Data Collection

All subjects responded to a general questionnaire including socio-economic and lifestyle aspects of the mother. Self-reported weight at delivery, number of gestational weeks at delivery, and delivery method were also recorded. Additionally, a physical examination evaluated basic anthropometric parameters (height, weight, mid-arm circumference) blood pressure, and hemoglobin. Data collection was done through face-to-face interviews the day of HM sample collection. In addition, the date of birth and gender information of the baby was collected after the data collection, since the data was not included in the initial questionnaires. Subjects were contacted by phone and were asked to clarify these two aspects retrospectively. 

### 2.5. HM Sampling

**Figure 1:**
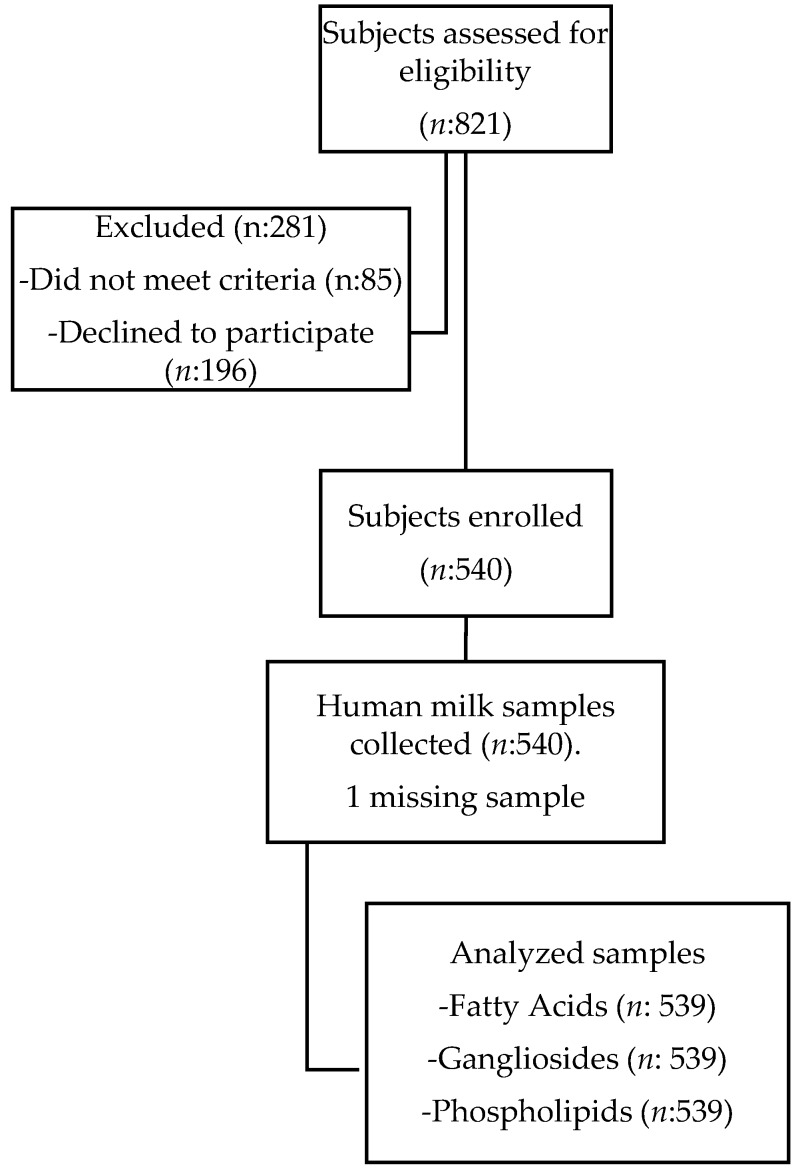
Study flowchart for subject recruitment.

Breast milk sampling was standardized for all subjects and an electric pump (Horigen HNR/X-2108ZB, Xinhe Electrical Apparatuses Co., Ltd., Beijing, China) was used to sample the milk. Samples were collected at the second feeding in the morning (9:00–11:00 a.m.) to avoid circadian influence on the outcomes. A single full breast was emptied and aliquots of 10 mL for colostrum and 40 mL for the remaining time points was secured for characterization purposes. The rest of the milk was returned to the mother for feeding to the infant. Each sample was distributed in freezing tubes, labelled with subject number, and stored at −80 °C until analysis. Figure 1 shows the study flowchart for the subjects’ recruitment.

### 2.6. Analytical Methods

#### 2.6.1. FA Quantification

FA profile was determined by preparing the methyl esters of FA (FAMEs). A direct transesterification of HM was performed with methanolic chloridric acid solution, as described by Cruz-Hernandez et al. [27]. Briefly, into a 10 mL screw cap glass test tube, milk (250 μL) was added and mixed with 300 μL of internal standard FAME 11:0 solution (3 mg/mL) and 300 μL of internal standard TAG 13:0 solution (3 mg/mL). After addition of 2 mL of methanol, 2 mL of methanolic chloridric acid (3 N), and 1 mL of hexane, the tubes were heated at 100 °C for 90 min. To stop the reaction 2 mL of water were added and after centrifugation (1200× *g* for 5 min) the upper phase (hexane) was transferred into gas chromatography vials. The analysis of FAMEs was performed by GC using a CP-Sil 88 capillary column (100 m, 0.25 mm i.d. 0.25 µm film thickness) and their identification by comparison of retention time with authentic standards (GC standard Nestlé 36 from NuCheck-Prep, Elysan, MN, USA). 

#### 2.6.2. Phospholipid Quantification

PL were quantified as previously described by Giuffrida et al. [28]. Briefly, 250 mg of maternal milk was mixed with 250 mg of water and 9.5 mL of chloroform/methanol (2/1 *v*/*v*). After addition of 10 µL of phosphatydilglycerol internal standard solution (5 mg/mL), the sample solution was put into an ultrasonic bath at 40 °C for 15 min. After centrifugation (1000 relative centrifugal force (RCF), for 10 min), the sample solution was filtered through 0.2 µm PTFE filters; the filtrate was mixed with 2 mL of potassium chloride solution (8.8 g/L) and centrifuged (1000 RCF for 10 min). The organic phases were evaporated to dryness and the residual lipids were redissolved in 150 µL of chloroform/methanol (9/1 *v*/*v*), filtered through 4 mm polyvinylidene fluoride (PVDF) membrane filters analyzed by high performance liquid chromatography coupled with evaporative light scattering detector (HPLC-ELSD). PL classes were separated by normal-phase HPLC using 2 Nucleosil 50-5, 250 × 3 mm, 5 µm (Macherey-Nagel, Easton, PA, USA) equipped with pre-column Nucleosil 50-5, 8 × 3 mm, 5 µm (Macherey-Nagel, Easton, PA, USA). All chromatography was performed at 55 °C. Solvent A contained ammonium formiate 3 g/L and solvent B of acetonitrile/methanol (100/3 *v*/*v*). Gradient conditions for PL analysis were as follows: time = 0 min 1% solvent A; time = 19 min 30% solvent A; time = 21 min 30% solvent A; time = 24 min 1% solvent A; with a flow rate 1 mL/min. Injection volume was 0.01 mL. The best signal and resolution was achieved at the following ELSD conditions: evap. = 90 °C; neb = 40 °C, flow rate of N_2_ = 1 L/min.

#### 2.6.3. Gangliosides Quantification

GD were quantified as previously described by Giuffrida et al. [2]. Briefly, HM (0.2 mL) was dissolved in water (1 mL) and mixed with 4 mL methanol/chloroform (2/1). After centrifugation (3000× *g*, for 10 min), the upper liquid phase was quantitatively transferred into a 15 mL centrifuge tube. The residue was mixed with water (1 mL), 2 mL of methanol/chloroform (2/1), shaken, put into an ultrasonic bath at 25 °C for 10 min, centrifuged (3000× *g*, for 10 min), and upper liquid phases polled together; the volume was adjusted to 12 mL with methanol 60% and pH to 9.2 by adding Na2HPO4 30 mmol/L (0.2 mL). The extract solution was loaded on an Oasis HLB VAC RC SPE cartridges (30 mg, 15 mL, Waters) previously conditioned with methanol (2 mL) and methanol 60% (2 mL). The sample was passed through the cartridge at maximum flow rate 2–3 mL /min. The sorbent was washed with 2 mL of methanol 60% and dried by vacuum suction for a few seconds; the analyte was eluted with methanol (2 mL). Solvent was evaporated to dryness under a nitrogen flow at 30 °C and the residual lipids were re-dissolved in 0.2 mL of methanol 70% and analysed by liquid chromatography (LC) coupled with quadrupole time of flight (QTOF), using an Aquity BEH C18 column (1.7 µm; 150 × 2.1 mm i.d.; Waters). All chromatography was performed at 50 °C. Solvent A was composed of water/methanol/ammonium acetate (1 mmol/L) (90/10/0.1 *v*/*v*/*v*) and solvent B of methanol/ammonium acetate (1 mmol/L) (100/0.1 *v/v*). Gradient conditions were as follows: time = 0 min 10% solvent A; time = 0.2 min 10% solvent A; time = 8.2 min 5% solvent A; time = 12.2 min 5% solvent A; time = 12.4 min 0% solvent A; time = 18.4 min 0% solvent A; time = 18.6 min 10% solvent A; time = 21 min 10% solvent A. Flow rate was 0.2 mL/min. Injection volume was 0.01 mL for GD3 and 0.005 mL for GM3. The mass spectrometer was equipped with an electrospray ionization (ESI) ion source. The ESI mass spectra were recorded in the negative ion mode under the following conditions: ion spray voltage (IS) −4000 V, temperature of the source 400 °C, declustering potential (DP) −40 V, ion source gases one and two at 40 and 35 psi, respectively, curtain gas at 15 psi, collision energy −40 V. GD3 and GM3, were monitored by transitions of the precursor ions to the *m*/*z* 290. Quantification was performed by the standard addition method.

## 3. Results

### 3.1. Demographics and Anthropometrics of Study Subjects 

In the current study we analyzed HM from 539 mothers (Figure 1), collected in a cross-sectional design over eight months postpartum. Milk obtained for analyses was a single, whole breast milk sample to have a comprehensive view on nutrient content. The details of the demographics and anthropometrics of the study subjects are outlined in Table 1. Groups of mothers, which delivered either a male or a female infant, were comparable for their age and anthropometric and demographic characteristics. Gestational age at birth (average 39 weeks) were also comparable between groups. The details of demographics and anthropometrics of the study subjects for the time period 0–4 days are not available.

### 3.2. FA

FA were determined by gas chromatography coupled with flame ionization detector (GC-FID), as previously described by Cruz-Hernandez et al. [27] and the results are listed in Table 2.

In our study total SFA content increased significantly from colostrum (35.7% of total FA) to transitional milk (38.9% of total FA) and decreased in mature milk (36.2% of total FA), with palmitic acid (16:0) being the most abundant FA and decreasing significantly (*p* < 0.05) from 23.2% in colostrum to 19.8% of total FA in mature milk (Table 2). Stearic acid (18:0) content was constant along the lactation period, i.e., colostrum, transitional, and mature milk, at about 5% of total FA, and medium-chain (MC) FA (10:0–14:0) content was low in colostrum (6.8% of total FA) compared to transitional (13.1% of total FA), and mature milk (11.0% of total FA) (Table 2). Arachidic (20:0) and lignoceric acids (24:0) were constant along the lactation time at about 0.2 and 0.1% of total FA, respectively. No significant differences (*p* > 0.05) on total SFA content were observed among cities in colostrum, and transitional milk (Table 2). SFA content was significant lower (*p* < 0.05) in mature milk from Suzhou (34.5% of total FA). Palmitic (22.5%, 19.4%, and 18.5% of total FA in colostrum, transitional, and mature milk, respectively) and stearic (4.9%, 4.5%, and 4.8% of total FA in colostrum, transitional and mature milk, respectively) FA also showed the lowest content in mature milk from Suzhou.

In the total population the MUFA content of HM decreased from 40.7% in colostrum to 36.9% of total FA in mature milk, with oleic acid (18:1*n*-9) being the most abundant FA and decreasing along the lactation time from 34.2% in colostrum to 31.9% of total FA in transitional and mature milk. Other MUFA (i.e., 17:1*n*-7, 20:1*n*-9, 22:1*n*-9. and 24:1*n*-9) also decreased over the lactation period (Table 2). The highest level of total MUFA content was found in colostrum (43.1% of total FA), transitional (39.3% of total FA), and mature milk (38.3% of total FA) from Guangzhou (Table 2). The lowest level of total MUFA content was found in colostrum (38.4% of total FA), transitional (34.7% of total FA), and mature milk (34.3% of total FA) from Beijing (Table 2). HM samples obtained from mothers in Guangzhou contained the highest level of Oleic acid whereas milk obtained from mothers in Beijing contained the lowest level, respectively: colostrum (37.1% vs. 32.6% of total FA), transitional (34.0% vs. 30.3% of total FA), and mature milk (33.4% vs. 30.1% of total FA).

In the total population, total PUFA *n*-6 increased from 21.7% in colostrum to 24.1% of total FA in mature milk with linoleic acid (18:2*n*-6) being the most abundant FA and increasing along the lactation time from 18.9% in colostrum to 22.8% of total FA in mature milk. ARA (20:4*n*-6) content decreased from 0.9% to 0.5% of total FA from colostrum to mature milk. Beijing and Suzhou showed higher total PUFA*n*-6 content in colostrum (23.3% and 22.8% of total FA, respectively), transitional (22.5% and 22.9% of total FA, respectively), and mature milk (26.6% and 25.3% of total FA, respectively) than Guangzhou (Table 2). 

Total PUFA *n*-3 in HM from total population slightly increased from 1.4% in colostrum to 1.9% of total FA in mature milk with linolenic acid (18:3*n*-3) being the most abundant and increasing along the lactation time from 0.9% in colostrum to 1.5% of total FA in mature milk. DHA (22:6*n*-3) slightly decreased over lactation period from 0.5% in colostrum to 0.3% of total FA in mature milk, and EPA (20:5*n*-3) was present in a small amount (<0.1% of total FA in colostrum, transitional, and mature milk). The highest level of total PUFA *n*-3 content was found in colostrum (1.8% of total FA), transitional (2.1% of total FA), and mature milk (2.4% of total FA) from Suzhou (Table 2), which, as a consequence, showed the lowest *n*-6 to *n*-3 ratio (12.7% in colostrum, 10.9%in transitional milk, and 10.5% of total FA in mature milk). 

### 3.3. Phospholipids

PL classes were determined by LC-ELSD, as previously described by Giuffrida et al. [28] and the results are listed in Table 3.

We did not measure minor constituents, such as lysophosphatidylcholine, which may contribute only to small amounts of the infant’s diet.

**Figure 2:**
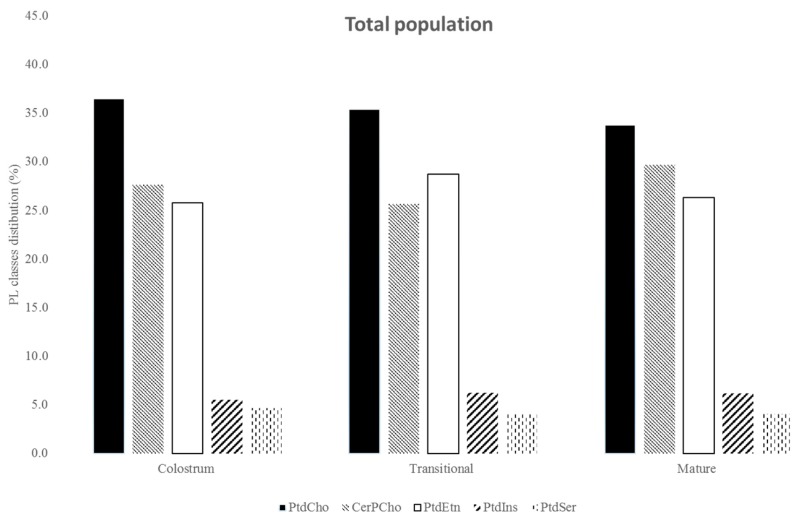
Change in phospholipid (PL) classes distribution in colostrum, transitional and mature milk.

From the total population, total PL content in HM decreased along the lactation period from 33.0 in colostrum to 24.2 mg/100 mL in mature milk, being significant lower (*p* < 0.05) in mature milk (Table 3). PtdCho was the most abundant PL in HM (from 12.0 mg/100 mL in colostrum to 8.2 mg/100 mL in mature milk) followed by CerPCho (from 9.1 mg/100 mL in colostrum to 7.2 mg/100 mL in mature milk), PtdEtn (from 8.5 mg/100 mL in colostrum to 6.4 mg/100 mL in mature milk), PtdIns (from 1.8 mg/100 mL in colostrum to 1.5 mg/100 mL in mature milk), and PtdSer (from 1.5 mg/100 mL to 1.0 mg/100 mL in mature milk). The PL class distribution was similar in colostrum, transitional, and mature milk (Figure 2).

**Figure 3:**
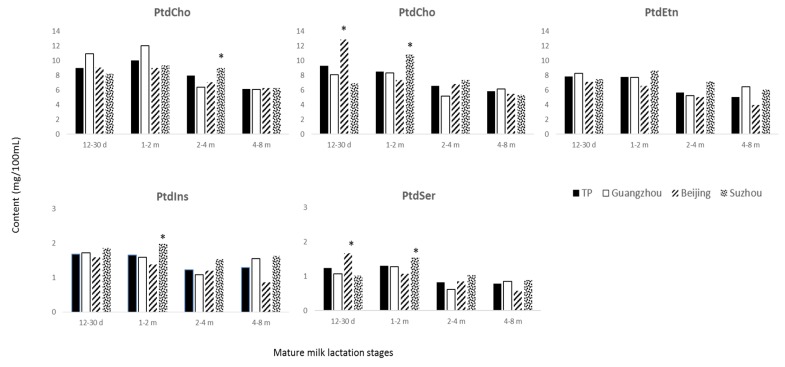
PL contents at different lactation stages, i.e., 12–30 days, 1–2 months, 2–4 months, and 4–8 months postpartum are shown in the total population and cities. TP stands for total population. * indicates significant difference (*p* < 0.05) among cities within the lactation stage.

Among the cities, PtdCho content did not show significant difference (*p* > 0.05) (Table 3); however, when considering the mature milk data at different lactation stages (Figure 3), PtdCho content was significant higher at 2–4 months in Suzhou (9.0 mg/100 mL) when compared to Beijing (7.1 mg/100 mL) and Guangzhou (6.4 mg/100 mL). CerPCho content was significant higher (*p* < 0.05) in colostrum (10.9 mg/100 mL) of lactating mothers from Beijing and in transitional milk (8.5 mg/100 mL) of lactating mothers from Suzhou (Table 3); when considering the different lactation stages of mature milk (Figure 3), Beijing showed significantly higher content (12.9 mg/100 mL) at 12–30 days and Suzhou at 1–2 months (10.9 mg/100 mL). PtdEtn content was significant lower (*p* < 0.05) in colostrum (7.6 mg/100 mL) and mature milk (5.3 mg/100 mL) of lactating mother from Beijing and significant higher (*p* < 0.05) in colostrum (12.6 mg/100 mL) and transitional milk (10.8 mg/100 mL) of lactating mothers from Suzhou (Table 3); when considering the mature of the milk data at different lactation stages (Figure 3) Suzhou showed the highest contents of PtdEtn at 1–2 months (8.6 mg/100 mL). PtdIns content was significant low (*p* < 0.05) in mature milk (1.2 mg/100 mL) of lactating mothers from Beijing and significant higher (*p* < 0.05) in colostrum (2.3 mg/100 mL), transitional (2.4 mg/100 mL), and mature milk (1.7 mg/100 mL) of lactating mothers from Suzhou (Table 3); within mature milk (Figure 3) Suzhou showed the highest content of PtdIns (2.0 mg/100 mL) at 1–2 months. PtdSer content was significant higher (*p* < 0.05) in colostrum (1.8 mg/100 mL) of lactating mothers from Beijing and significantly different (*p* < 0.05) in transitional (1.3 mg/100mL) and mature milk (1.2 mg/100 mL) of lactating mothers from Suzhou (Table 3). Within mature milk (Figure 3) Beijing showed the highest PtdSer content (1.7 mg/100 mL) at 12–30 days and Suzhou at 1–2 months (1.5 mg/100 mL, respectively). Finally, Suzhou showed significant higher (*p* < 0.05) PL content in colostrum (38.9 mg/100 mL), transitional milk (34.9 mg/100 mL), and mature milk (26.0 mg/100 mL), and Beijing showed the lowest content in mature milk (22.3 mg/100 mL) (Table 3).

### 3.4. Gangliosides

Gangliosides were determined by LC-MS/MS as described by Giuffrida et al. [29] and the results are listed in Table 4.

From the total population, the amount of GD changed during the lactation period (Table 4), with GM3 significantly increasing (*p* < 0.05) from 3.8 mg/mL in colostrum to 10.1 mg/L in mature milk and GD3 significantly decreasing (*p* < 0.05) from 4.1 mg/mL in colostrum to 1.0 mg/mL in mature milk. Total gangliosides increased significantly (*p* < 0.05) from 8.0 mg/L in colostrum to 11.0 mg/L in mature milk (Table 4). However, variability was high and total ganglioside content ranged from 1.66–28.44 mg/L in colostrum, 2.77–22.04 mg/L in transitional milk, and between 0.90–36.88 mg/L in mature milk; GM3 contents ranged between 0.63–13.03 mg/L in colostrum, 1.01–17.71 mg/L in transitional milk, 3.45–25.97 mg/L at 1–2 months, 3.45–25.97 mg/L at 2–4 months, and between 5.17–34.41 mg/L at 4–8 months; GD3 contents ranged between 0.55–18.04 mg/L in colostrum, 0.06–15.52 mg/L in transitional milk, 0.15–4.93 mg/L at 1–2 months, 0.06–5.0 mg/L at 2–4 months, and between 0.05 and 6.77 mg/L at 4–8 months. The GM3/GD3 ratio also increased over the lactation period, to 0.9 in colostrum and 10.1 in mature milk, consistent with the variation of GM3 and GD3 described above.

**Figure 4:**
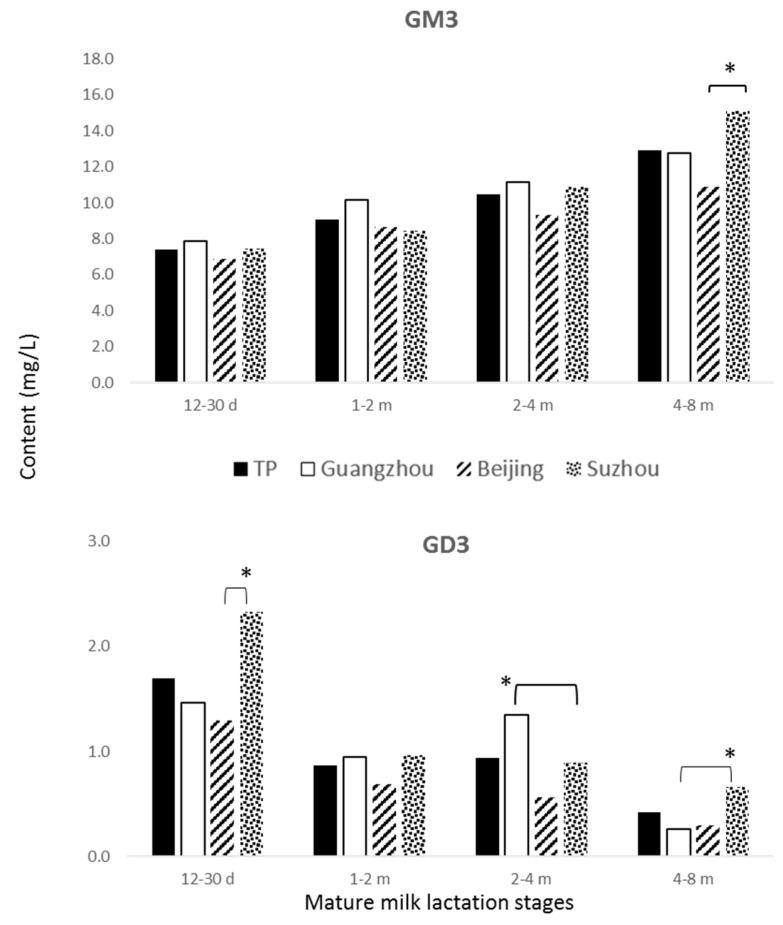
GM3 and GD3 contents at different mature milk lactation stages, i.e., 12–30 days, 1–2 months, 2–4 months, and 4–8 months postpartum are shown in total population (TP) and cities. * stands for significant difference (*p* < 0.05) at 4–8 months between Beijing and Suzhou for GM3, at 12–30 days between Beijing and Suzhou, and at 2–4 months and at 4–8 months between Guangzhou and Suzhou for GD3.

Among the different cities, GM3 content was comparable (*p* > 0.05) in colostrum; GM3 highest content (*p* < 0.05) in transitional milk (7.7 mg/L) was observed in HM of lactating mothers from Guangzhou and in mature milk in lactating mothers from Guangzhou and Suzhou, at 10.5 and 10.8 mg/L, respectively (Table 4). Within mature milk (Figure 4) Beijing, Guangzhou, and Suzhou showed the highest GM3 content at 4–8 months (11.0 ± 3.9, 12.3 ± 5.5, and 15.6 ± 6.1 mg/L, respectively). The highest content (*p* < 0.05) of GD3 was observed in colostrum of lactating mothers from Suzhou (8.6 mg/L); GD3 content was comparable (*p* > 0.05) in transitional milk among the different cities and between Guangzhou and Suzhou in mature HM (Table 4). However, when considering mature milk at different lactation stages (Figure 4), Beijing, Guangzhou, and Suzhou showed the highest GD3 content at 12–30 days (0.9 ± 1.3, 1.1 ± 1.1, and 1.5 ± 2.2 mg/L, respectively). Suzhou showed the highest content (*p* < 0.05) of total GD in colostrum and mature milk (12.6 and 11.9 mg/L, respectively), the highest content (*p* < 0.05) of total GD in transitional milk was observed in Guangzhou (10.7 mg/L) (Table 4).

## 4. Discussion

This study measured the FA, PL, and GD content and the profile of 539 HM samples from Beijing, Guangzhou, and Suzhou.

### 4.1. FA

Results from the total population (Table 2) showed a total SFA content of 35.7% ± 3.9% in colostrum, of 38.9% ± 4.1% in transitional milk, and of 36.2% ± 4.7% in mature milk. Chinese studies have reported SFA level in colostrum ranging from 36.8% to 41.3% [30,31], in transitional milk from 35.2% to 42.6% [31,32] and in mature milk from 35.1% to 41.1% [30,31,32,33,34], in agreement with our results. When considering other populations (e.g., Caucasian, American) the SFA level in colostrum was 42.3%–43.7% [35,36,37], in transitional milk it ranged from 43.1% to 45.2% [36,37] and, in mature milk, from 37.4% to 57.1% [34,35,36,37,38], therefore, Chinese populations seem to show lower amount of total SFA in colostrum, transitional and mature milk when compared to other populations. 

In this study, main SFA, lauric (12:0), myristic (14:0), palmitic (16:0), and stearic (18:0) acid contents were 2.6% ± 1.6%, 3.8% ± 1.7%, 23.2% ± 1.9%, and 5.2% ± 1.0% of total FA, respectively, in colostrum (Table 2); 6.1% ± 2.3%, 5.5% ± 2.2%, 20.5% ± 2.3%, and 5.0% ± 0.8% of total FA in transitional milk (Table 2); and 5.2% ± 1.9%, 4.2% ± 1.7%, 19.8% ± 2.6%, and 5.1% ± 1.1% of total FA, respectively, in mature milk (Table 2). Among the Chinese population, lauric, myristic, palmitic, and stearic acids ranged between 3.0%–4.9%, 5.2%–5.3%, 20.1%–23.3%, and 6.0%–7.0% of total FA, respectively, in colostrum [30,31]; between 4.2%–6.5%, 3.8%–6.4%, 19.7%–23.3%, and 5.4%–8.1% of total FA, respectively, in transitional milk [31,32]; and finally between 3.8%–6.3%, 3.4%–6.5%, 17.3%–22.3%, and 5.0%–8.0% of total FA, respectively, in mature milk [30,31,32,33,34]. When considering other populations, lauric, myristic, palmitic, and stearic acids ranged between 1.2%–4.5%, 4.8%–7.3%, 24.0%–27.3%, and 5.5%–7.1% of total FA, respectively, in colostrum [35,36,37]; between 5.2%–6.5%, 6.5%–7.7%, 22.2%–22.6%, and 5.7%–7.4% of total FA, respectively, in transitional milk [36,37]; and finally between 3.7%–6.1%, 4.9%–7.0%, 18.7%–23.0%, and 4.8%–7.6% of total FA, respectively, in mature milk [34,35,36,37,38]. Philippian population showed high lauric (13.82%) and myristic (12.12%) FA contents [34] and it was reported [20,21] that 10:0, 12:0, and 14:0 FA content increases when lactating women consumed high-carbohydrate diets, whereas the secretion of the 18-carbon chain unsaturated FA, which are derived from the diet, decreased. High contents of lauric (10.2%) and myristic FA (9.1%) have been also reported in the milk of women from Nigeria [4] as a typical response to a high-carbohydrate diet. 

Results from the total population (Table 2) showed total MUFA content of 40.7% ± 3.8% in colostrum, of 37.7% ± 4.3% in transitional milk, and of 36.9% ± 4.1% in mature milk. Chinese studies have reported MUFA levels in colostrum ranging from 34.7% [30] to 43.1% [31], in transitional milk from 30.8% to 42.9% [31,32], and in mature milk from 28.5% to 45.6% [30,31,32,33,34], in agreement with our results.

When considering other population, MUFA levels in colostrum ranged between 32.1%–44.4% of total FA [35,36,37], in transitional milk it was 35.1% of total FA [37], and in mature milk ranged from 30.3%–44.4% of total FA [34,35,37,38], therefore, Chinese populations seem to show comparable MUFA content in colostrum, transitional and mature milk to other populations.

Among MUFA, oleic acid was the most abundant FA and its content ranged from 34.4% in colostrum to 31.9% in transitional and mature milk. In the Chinese population oleic acid ranged from 28.4%–36.3% of total FA in colostrum [30,31]; from 25.9%–36.5% of total FA in transitional milk [31,32], and from 24.9%–38.1% in mature milk [30,31,32,33,34], and in other populations from 28.4%–40.1%, from 27.7%–32.1%, and from 21.9%–40.5% of total FA in colostrum [35,36,37], transitional [36,37], and mature milk [34,35,36,37,38], respectively.

Results from the total population (Table 2) showed total *n*-6 and *n*-3 PUFA content of 21.7 ± 3.6 and 1.4% ± 0.5% in colostrum, respectively, of 21.6 ± 3.8 and 1.9% ± 0.7% in transitional milk, and of 24.1 ± 5.0 and 1.9% ± 0.9% of total FA in mature milk. Chinese studies have reported PUFA levels in colostrum ranging from 14.8%–22.5% for *n*-6PUFA and from 2.9%–3.9% for *n*-3PUFA [30,31], in transitional milk from 13.7%–27.6% for *n*-6PUFA and from 2.5%–5.1% for *n*-3PUFA [31,32], and in mature milk from 14.1%–27.8% for *n*-6PUFA and from 2.6%–6.8% for *n*-3PUFA [30,31,32,33,34], therefore, the values are in agreement with the *n*-6PUFA results of this study, but higher for *n*-3PUFA.

When considering other populations, PUFA levels in colostrum ranged between 11.2%–14.0% for *n*-6PUFA and from 1.9%–3.5% of total FA for *n*-3PUFA [35,36,37], in transitional milk from 12.3%–14.1% for *n*-6PUFA and from 1.5%–3.3% of total FA for *n*-3PUFA [36,37], and in mature milk from 9.5%–20.3% for *n*-6PUFA and from 1.3%–3.2% of total FA for *n*-3PUFA [34,35,36,37,38]. Therefore, the Chinese populations seem to show higher contents of total *n*-6 PUFA when compared to other populations.

Among PUFA, LA (18:2*n*-6), and ALA (18:3*n*-3), considered essential FA because humans lack the enzymes required for their biosynthesis, were the most abundant FA we observed in colostrum, mature and transitional milk (18.9% ± 3.6%, 19.7% ± 3.8%, and 22.8% ± 4.9% of total FA for LA, respectively and for 0.9% ± 0.4%, 1.4% ± 0.6%, and 1.5% ± 0.9% of total FA for ALA, respectively). 

In the Chinese population, LA and ALA ranged from 10.3%–19.2% and from 0.9%–1.3% of total FA, respectively, in colostrum [30,31,32]; from 9.8%–23.3% and from 0.9%–2.2% of total FA, respectively, in transitional milk [31,32]; and from 10.9%–23.7% and from 0.9%–3.0% of total FA, respectively, in mature milk [30,31,32,33,34], in agreement with our findings. 

In other populations, LA and ALA ranged from 8.6%–11.9% and from 0.7%–1.1% of total FA, respectively, in colostrum [35,36,37]; from 10.3%–12.5% and from 0.8%–1.3% of total FA, respectively, in transitional milk [36,37]; and from 7.9%–17.8% and from 0.4%–1.4% of total FA, respectively, in mature milk [34,35,36,37,38].

Finally, in our study, DHA contents for the total population ranged from 0.3%, in mature milk, to 0.5% in colostrum and transitional milk, therefore, lower than the DHA content reported for Chinese marine populations in colostrum (1.5%) [30], transitional (0.6%) [31], and mature milk (0.5%–2.8%) [30,33]. The ratio ARA/DHA (1.8–2.2) was comparable to average worldwide ratio of about 1.5 [22].

Among different cities, over lactation time, HM from Beijing showed slightly higher SFA content (Table 2), Guangzhou the highest MUFA content (Table 2), and Suzhou the highest *n*-3PUFA content (Table 2).

It is known that the type of fat/oil in the maternal diet influences the FA composition of breast milk. Francois et al. [22] showed that the consumption of six different dietary fats, each providing a specific FA, caused an acute response in HM FA composition, especially within 24 h, and that the response remained significantly elevated for 1–3 days after consumption of dietary fat. Therefore, difference observed in HM FA composition may reflect variation in maternal diet [33].

However, a careful analyses of dietary habits of Guangzhou, Beijing, and Suzhou needs to be performed for correlating to HM composition. 

### 4.2. Phospholipids

Several studies have recognized the importance of PL for infant growth [39,40,41]. At the same time, PL are involved in immunity and inflammatory responses [42], and in neuronal signaling [43].

PL content in HM significantly (*p* < 0.005) decreased along the lactation period from 33.0 in colostrum to 24.2 mg/100 mL in mature milk, in agreement with previous studies performed elsewhere [12,44]. The PL class distribution was similar in colostrum, transitional and mature milk (Figure 2).

PL as emulsifiers are essential for the solubilization of dietary fats and as a consequence for their digestion and absorption. In this regard, the higher content of PL in colostrum and transitional HM compared to mature milk might explain the good fat absorption from HM by the newborn, despite poor pancreatic secretion, as suggested by Harzer et al. [11]. A decrease in PL content in HM along the lactation stage might occur because the diameter of the milk fat globule membrane increases [11,45], decreasing the PL/TAG ratio [7,8].

Our study showed that PtdCho was the most abundant PL in HM (Figure 2), followed by CerPCho and PtdEtn, and PtdIns and PtdSer, in agreement with previous studies [3,11,12,44,46]. PtdCho and CerPCho are important sources of choline considered as an essential nutrient for infants. Choline is a precursory amino alcohol of the neurotransmitter acetylcholine, it acts by regulating the transduction signal, and serves as a source of methyl groups in intermediate metabolism, being considered essential for optimum development of the brain [7,8]. In addition, CerPCho can reduce cholesterol absorption between 20.4%–85.5%, depending on the ingested dose (0.1% and 5.0%, respectively) [47], being possibly involved in cholesterol regulation programming. 

The amount of total PL in colostrum (33.0 ± 13.2 mg/100 mL), transitional (28.5 ± 14.4 mg/100 mL), and mature milk (24.2 ± 11.4 mg/100 mL), was comparable to the values reported by Bitman et al. [44] (35, 31, and 27 mg/100 mL, respectively), Thakkar et al. [48] (20.8–24.2 mg/100 mL in mature milk), and Garcia et al. [49] (15.3–47.4 mg/100 mL in mature milk); higher than the values reported by Sala-Vila et al. [12] (13.5, 14.0, and 9.8 mg/100 mL, respectively), Lopez et al. [50] (13.5 mg/100 mL in mature milk) and Zou et al. [51] (16.8, 22.3, and 19.2 mg/100 mL, respectively). 

In the total population PtdCho contents in colostrum, transitional, and mature milk were 12.0 ± 5.8, 10.1 ± 5.5, and 8.2 ± 5.0 mg/100 mL, respectively, comparable to values reported in literature, 4.3–11.2, 5.7–9.4, and 2.0–11.2 mg/100 mL, respectively [44,48,49,50,51,52,53,54,55,56]. 

CerPCho contents in colostrum, transitional, and mature milk were 9.1 ± 4.0, 7.3 ± 4.1, and 7.2 ± 4.0 mg/100 mL, respectively, comparable to values reported in the literature of 5.3–11.0, 9.0–11.6, and 3.1–13.5 mg/100 mL, respectively [44,48,49,50,51,52,53,54,55,56,57].

PtdEtn contents in colostrum, transitional, and mature milk were 8.5 ± 5.2, 8.2 ± 5.3, and 6.4 ± 3.4 mg/100 mL, respectively, higher than values reported in the literature for colostrum and transitional milk 1.4–6.4 and 1.5–5.6 mg/100 mL, respectively [44,51], and comparable to values reported for mature milk of 0.2–8.1 mg/100 mL [3,11,12,44,48,49,51,52].

PtdIns and PtdSer contents in colostrum (1.8 ± 0.7 and 1.5 ± 1.6 mg/100 mL, respectively), transitional (1.8 ± 1.0 and 1.1 ± 0.8 mg/100mL, respectively), and mature milk (1.5 ± 0.7 and 1.0 ± 1.1 mg/100 mL, respectively) were comparable to values reported in previous studies for PtdIns, 1.4–3.3, 1.5–2.2, and 0.2–2.2 mg/100 mL, respectively, and for PtdSer, 2.1–3.6, 1.5–2.2, and 0.8–4.5 mg/100 mL [44,48,49,50,51,53].

Among the different cities, Suzhou showed the highest total PL and PtdEtn levels in colostrum, transitional, and mature milk (Table 3). Dietary sources of PtdEtn may be lecithin from rapeseed oil, whose consumption may explain also the higher content of ALA in HM from Suzhou. However, a careful analyses of dietary habits of this region needs to be performed for correlating to HM composition.

It is well known [58] that lipid and liposoluble nutrients content increases towards the latter part of a feeding session, a phenomenon that has been corroborated by biochemical analyses of total milk fat in fore-milk, and hind-milk [59,60]. Therefore, in order to assure sample homogeneity in our study all efforts have been made to collect fully-expressed milk. Among the cited studies, only Bitman et al., Thakkar et al., Holmes et al., and Fischer et al. [44,48,54,56] refer to full breast milk samples, Sala-Vila et al. [12] to fore-milk, and no detailed sampling procedure is described in the other studies. Analysis performed in fore-milk and hind-milk rather than fully-expressed milk could explain the discrepancy among results.

### 4.3. Gangliosides

GD are widely distributed in almost all human tissues, with the highest amount found in neural tissue and extra-neural organs, such the lung, spleen, and gut. It has been reported that during the first stages of life, dietary GD may have an important role in preventing infections [61] and in cognitive development functions [10,62].

Our data confirmed, as previously reported [14,16,17,29,63,64], that the amount of GD changes during the lactation period, with GD3 decreasing and GM3 increasing over the lactation period. Rueda et al. [15] postulated that a high concentration of GD3 in early milk may reflect its biological role in the development of organs, such as the intestine, as was observed in our study in all cities. The increase in GM3 in mature milk has been associated with signal transduction, cell adhesion, and growth factor receptors, leading to the development of the immune and central nervous systems [14,17,61]. In the studied population, the sum of GM3 and GD3 increased from 8.0 mg/L in colostrum to 11.0 mg/L in mature milk, as previously published [29]. It has been reported [64] that the sum of GM3 and GD3 can range from as low as 2 mg/L to as high as 25 mg/L, depending on breast milk sampling, population demographics, diet, and analytical methodologies. In this study, total ganglioside content ranged from 1.66–28.44 mg/L in colostrum, 2.77–22.04 mg/L in transitional milk, and between 0.90–36.88 mg/L in mature milk, covering total GD contents previous reported, i.e., 2.8–59.7 mg/L in colostrum [14,15,17,18,29,63,65], 0.9–30.7 mg/L in transitional [14,15,17,18,63,65], and 1.6–68.6 mg/L in mature milk [14,15,17,18,29,48,63,65,66]. When considering average values, in colostrum and transitional milk, GM3 content (3.8 and 5.5 mg/L, respectively) was lower than the one reported by Ma et al. [63] (6.5–7.1 and 8.3–9.6 mg/L, respectively). Within mature milk, at 1–2 months GM3 content (9.08 mg/L) was comparable to the one reported by Ma et al. [63,64] (8.3–11.3 mg/L) and higher than the content reported by Thakkar et al. [48] (2.3–2.9 mg/L); after 3–8 months GM3 content (10.46–12.92 mg/L) was lower than what reported by Ma et al. [63,64] (17.4–21.4 mg/L) and higher than the content reported by Thakkar et al. [48] (3.9 mg/L). However, when considering minimum and maximum values, GM3 contents (0.63–13.03, 1.01–17.71, and 0.8–34.41 mg/L, in colostrum, transitional, and mature milk, respectively) were comparable with GM3 contents previously reported [48,63,64]. 

As for GM3, when considering average values, in colostrum and transitional milk, GD3 content (4.1 and 3.0 mg/L, respectively) was lower than the one reported by Ma et al. [63] (20 and 10 mg/L, respectively). Within mature milk, at 1–2 months GD3 content (0.87 mg/L) was lower than the one reported by Ma et al. [63,64] (4.6–7.0 mg/L) and by Thakkar et al. [48] (1.9–2.3 mg/L); after 3–8 months GD3 content (0.25–0.50 mg/L) was lower than that reported by Ma et al. [63,64] (1.5–2.7 mg/L) and by Thakkar et al. [48] (1.7 mg/L). However, when considering minimum and maximum values, GD3 contents (0.6–18.0, 0.1–15.5, and 0.1–9.3 mg/L, in colostrum, transitional, and mature milk, respectively) were comparable with GD3 contents previously reported [48,63,64]. Among the cities Suzhou showed the highest GM3 and GD3 contents (Table 4) in colostrum and mature milk.

Ma et al. [64] suggested that the ganglioside concentrations in HM at any time point may be influenced by the mother's dietary intake of gangliosides or their precursors. It was demonstrated [67] that GD3 and GM3 are transferred across the human placenta using an ex vivo model of dually-perfused isolated human placental lobules, suggesting that they are available to the developing fetus. Therefore, a careful analysis of dietary habits in this region needs to be performed for correlating to HM GD composition.

## 5. Conclusions

In this study, FA, PL, and GD contents and compositions of HM from lactating women living in Suzhou, Guangzhou, and Beijing were evaluated.

HM was collected over a period of eight months, allowing the observation of lipid compositional changes during lactation.

SFA, MUFA, and PL content decreased during lactation, PUFA and GD content increased. Among different cities, over lactation time, HM from Beijing showed the highest SFA content, HM from Guangzhou showed the highest MUFA content, and HM from Suzhou showed the highest *n*-3PUFA content. The highest total PL and GD contents were observed in HM from Suzhou. In order to investigate the influence of the diet on maternal milk composition, a careful analysis of dietary habits of these population needs to be performed in future work.

## Figures and Tables

**Table 1:** Maternal descriptive characteristics.

	5–11 Days	12–30 Days	1–2 Months	2–4 Months	4–8 Months
	(*n* = 90)	(*n* = 90)	(*n* = 90)	(*n* = 90)	(*n* = 90)
**Mother**					
Age (years), Mean ± SD	27 ± 4	27 ± 3	28 ± 4	27 ± 4	26 ± 4
Natural delivery	27 ± 4	27 ± 3	28 ± 5	26 ± 4	26 ± 4
Caesarean delivery	28 ± 3	27 ± 4	29 ± 4	28 ± 4	27 ± 4
Height (cm), Mean ± SD	160 ± 4	160 ± 5	161 ± 5	161 ± 5	159 ± 5
Weight (kg), Mean ± SD	60.7 ± 8.7	60.8 ± 7.9	61.9 ± 8.9	58.4 ± 8.3	56.2 ± 8.1
BMI (kg/m^2^), Mean ± SD	23.7 ± 3.3	23.7 ± 2.8	23.9 ± 3.1	22.5 ± 2.9	22.2 ± 3.1
Gestational weight gain(kg), Mean ± SD	16.7 ± 7.4	16.2 ± 6.0	15.9 ± 5.7	15.9 ± 5.9	14.9 ± 7.6
Postpartum weight loss (kg), Mean ± SD	9.1 ± 6.1	8.6 ± 5.3	9.8 ± 4.0	10.0 ± 6.2	10.6 ± 5.9
Gestational age at birth (weeks), Mean ± SD	39.3 ± 1.2	39.2± 1.3	39.2 ± 1.6	39.4 ± 1.3	39.5 ± 1.5

SD: standard deviation.

**Table 2:** Median fatty acid composition of HM expressed as g/100 g of total FA.

FA (g/100 g)	Total Population			Guangzhou			Beijing			Suzhou		
	Colostrum (0–5 Days) *n* = 113	Transitional (6–15 Days) *n* = 81	Mature (16 Days–8 Months) *n* = 345	Colostrum (0–5 Days) *n* = 38	Transitional (6–15 Days) *n* = 22	Mature (16 Days–8 Months) *n* = 120	Colostrum (0–5 Days) *n* = 45	Transitional (6–15 Days) *n* = 21	Mature (16 Days–8 Months) *n* = 113	Colostrum (0–5 Days) *n* = 30	Transitional (6–15 Days) *n* = 38	Mature (16 Days–8 Months) *n* = 112
10:0	0.5 ± 0.4	1.5 ± 0.5 ^†^	1.6 ± 0.4 ^‡^	0.4 ± 0.4	1.4 ± 0.5	1.5 ± 0.5	0.6 ± 0.5	1.5 ± 0.4	1.6 ± 0.4	0.5 ± 0.4	1.6 ± 0.5	1.6 ± 0.4
12:0	2.6 ± 1.6	6.1 ± 2.3 ^†^	5.2 ± 1.9	2.3 ± 1.5	5.5 ± 2.1	5.0 ± 2.1	2.6 ± 1.7	6.5 ± 1.7	5.3 ± 1.6	2.7 ± 1.7	6.3 ± 2.6	5.3 ± 1.9
14:0	3.8 ± 1.7	5.5 ± 2.2 ^†^	4.2 ± 1.7 ^‡^	3.6 ± 1.8	5.2 ± 1.9	4.1 ± 2.0	3.8 ± 1.7	5.8 ± 1.4	4.3 ± 1.4	4.0 ± 1.7	5.2 ± 2.7	4.0 ± 1.7
16:0	23.2 ± 1.9	20.5 ± 2.3 ^†^	19.8 ± 2.6 ^‡^	23.9 ± 1.9	21.5 ± 2.1	20.6 ± 2.6	22.8 ± 2.2	21.5 ± 2.2	19.8 ± 2.2	22.5 ± 1.3	19.4 ± 2.2	18.5 ± 2.6
16:1*n*-7	2.0 ± 0.8	2.2 ± 0.7 ^†^	2.0 ± 0.6	1.7 ± 1.0	2.4 ± 0.8	2.2 ± 0.7	2.2 ± 0.6	1.7 ± 0.6	2.0 ± 0.5	1.8 ± 0.8	2.2 ± 0.5	2.0 ± 0.6
18:0	5.2 ± 1.0	5.0 ± 0.8	5.1 ± 1.1	5.5 ± 1.2	5.3 ± 0.7	5.4 ± 1.2	5.1 ± 0.9	5.4 ± 0.7	5.1 ± 1.0	4.9 ± 0.9	4.5 ± 0.8	4.8 ± 1.0
18:1*n*-9	34.2 ± 3.2	31.9 ± 3.6 ^†^	31.9 ± 3.6	37.1 ± 2.8	34.0 ± 2.2	33.4 ± 3.3	32.6 ± 2.9	30.3 ± 2.9	30.1 ± 2.9	34.0 ± 2.5	31.0 ± 4.1	31.7 ± 3.7
18:1*n*-7	2.5 ± 0.4	2.2 ± 0.5 ^†^	1.9 ± 0.3 ^‡^	2.7 ± 0.5	2.2 ± 0.4	2.0 ± 0.3	2.3 ± 0.4	2.0 ± 0.3	1.7 ± 0.2	2.4 ± 0.3	2.3 ± 0.6	1.9 ± 0.3
18:2*n*-6	18.9 ± 3.6	19.7 ± 3.8 ^†^	22.8 ± 4.9 ^‡^	15.7 ± 2.8	18.0 ± 3.4	19.7 ± 4.3	20.2 ± 3.5	20.2 ± 3.6	25.1 ± 3.9	19.9 ± 3.0	21.0 ± 3.9	23.8 ± 5.2
18:3*n*-3	0.9 ± 0.4	1.4 ± 0.6 ^†^	1.5 ± 0.9 ^‡^	0.7 ± 0.3	1.0 ± 0.5	1.0 ± 0.6	0.9 ± 0.4	1.1 ± 0.7	1.6 ± 1.1	1.2 ± 0.3	1.7 ± 0.6	2.0 ± 0.8
18:3*n*-6	0.05 ± 0.07	0.09 ± 0.06 ^†^	0.14 ± 0.06 ^‡^	<0.05	0.1 ± 0.1	0.1 ± 0.1	0.1 ± 0.1	0.1 ± 0.1	0.2 ± 0.1	<0.05	0.1 ± 0.1	0.1 ± 0.1
20:0	0.2 ± 0.1	0.2 ± 0.05	0.2 ± 0.1	0.2 ± 0.1	0.2 ± 0.1	0.2 ± 0.1	0.2 ± 0.1	0.2 ± 0.1	0.2 ± 0.1	0.2 ± 0.1	0.1 ± 0.1	0.2 ± 0.1
20:1*n*-9	0.9 ± 0.3	0.5 ± 0.2 ^†^	0.4 ± 0.2	1.0 ± 0.3	0.5 ± 0.2	0.4 ± 0.1	0.7 ± 0.3	0.5 ± 0.1	0.3 ± 0.1	0.9 ± 0.4	0.5 ± 0.2	0.6 ± 0.3
20:2*n*-6	1.2 ± 0.4	0.6 ± 0.3 ^†^	0.4 ± 0.1 ^‡^	1.1 ± 0.4	0.5 ± 0.3	0.4 ± 0.1	1.1 ± 0.4	0.8 ± 0.3	0.4 ± 0.1	1.3 ± 0.4	0.6 ± 0.2	0.4 ± 0.1
20:3*n*-6	0.7 ± 0.2	0.5 ± 0.2 ^†^	0.4 ± 0.1 ^‡^	0.6 ± 0.2	0.4 ± 0.1	0.3 ± 0.2	0.8 ± 0.3	0.6 ± 0.2	0.4 ± 0.1	0.7 ± 0.2	0.5 ± 0.1	0.4 ± 0.1
20:5*n*-3	0.04 ± 0.05	0.05 ± 0.06 ^†^	0.05 ± 0.07	<0.05	0.10 ± 0.1	<0.05	<0.05	<0.05	0.1 ± 0.1	0.1 ± 0.1	<0.05	0.1 ± 0.1
22:1*n*-9	0.2 ± 0.2	0.1 ± 0.1	0.1 ± 0.3	0.2 ± 0.1	0.1 ± 0.1	0.10 ± 0.2	0.2 ± 0.2	0.1 ± 0.1	0.1 ± 0.1	0.3 ± 0.3	0.1 ± 0.2	0.1 ± 0.5
20:4*n*-6 (ARA)	0.9 ± 0.3	0.7 ± 0.2 ^†^	0.5 ± 0.1 ^‡^	0.9 ± 0.2	0.7 ± 0.2	0.5 ± 0.2	1.1 ± 0.4	0.8 ± 0.2	0.5 ± 0.1	0.9 ± 0.2	0.7 ± 0.2	0.6 ± 0.1
24:0	0.2 ± 0.1	0.1 ± 0.1 ^†^	0.1 ± 0.1	0.2 ± 0.1	0.1 ± 0.1	0.1 ± 0.1	0.3 ± 0.2	0.1 ± 0.1	0.1 ± 0.1	0.2 ± 0.1	0.1 ± 0.1	0.1 ± 0.1
24:1*n*-9	0.4 ± 0.3	0.1 ± 0.1 ^†^	0.1 ± 0.1 ^‡^	0.4 ± 0.2	0.1 ± 0.1	0.1 ± 0.1	0.4 ± 0.3	0.1 ± 0.1	0.1 ± 0.1	0.4 ± 0.3	0.1 ± 0.1	0.1 ± 0.1
22:6*n*-3 (DHA)	0.5 ± 0.3	0.5 ± 0.2 ^†^	0.3 ± 0.2 ^‡^	0.7 ± 0.3	0.4 ± 0.4	0.3 ± 0.2	0.5 ± 0.2	0.5 ± 0.1	0.2 ± 0.1	0.5 ± 0.2	0.4 ± 0.2	0.3 ± 0.2
Total SFA	35.7 ± 3.9	38.9 ± 4.1 ^†^	36.2 ± 4.7 ^‡^	36.1 ± 4.0	39.2 ± 3.8	36.9 ± 4.8	35.4 ± 3.9	41.0 ± 3.0	36.4 ± 3.9	35.0 ± 3.7	37.2 ± 4.8	34.5 ± 4.9
Total MUFA	40.7 ± 3.8	37.7 ± 4.3 ^†^	36.9 ± 4.1	43.1 ± 3.3	39.3 ± 3.0	38.3 ± 3.6	38.4 ± 4.1	34.7 ± 3.5	34.3 ± 4.5	39.8 ± 2.6	36.2 ± 4.8	36.4 ± 4.4
MCFA	6.8 ± 2.4	13.1 ± 3.3	11.0 ± 2.6	6.3 ± 2.3	12.1 ± 3.5	10.6 ± 2.8	7.0 ± 2.2	13.8 ± 2.2	11.2 ± 2.3	7.2 ± 2.4	13.1 ± 3.8	10.9 ± 2.6
Total PUFA *n*-6	21.7 ± 3.6	21.6 ± 3.8	24.1 ± 5.0	18.3 ± 2.8	19.7 ± 3.4	21.1 ± 4.3	23.3 ± 3.6	22.5 ± 3.6	26.6 ± 3.9	22.8 ± 3.0	22.9 ± 3.9	25.3 ± 5.2
Total PUFA *n*-3	1.4 ± 0.5	1.9 ± 0.7	1.9 ± 0.9	1.4 ± 0.4	1.5 ± 0.6	1.3 ± 0.6	1.4 ± 0.4	1.6 ± 0.7	1.9 ± 1.1	1.8 ± 0.4	2.1 ± 0.6	2.4 ± 0.8
*n*-6 to *n*-3 ratio	14.4 ± 3.7	11.8 ± 3.7 ^†^	12.5 ± 5.5	13.1 ± 3.7	13.1 ± 3.9	16.5 ± 5.6	16.6 ± 3.0	14.1 ± 3.9	13.8 ± 5.6	12.7 ± 3.8	10.9 ± 2.9	10.5 ± 4.0
ARA to DHA ratio	1.8 ± 0.7	1.6 ± 0.5	2.2 ± 0.9 ^‡^	1.3 ± 0.5	1.8 ± 0.6	1.9 ± 1.0	2.2 ± 0.7	1.6 ± 0.5	2.3 ± 0.8	1.8 ± 0.4	1.8 ± 0.5	1.9 ± 0.8

FA, Fatty acids; ARA, arachidonic; DHA, docosahexaenoic; SFA, Saturated FA; MUFA, mono-unsaturated FA; MCFA, medium-chain FA; PUFA, polyunsaturated FA. * Values are presented as median ± standard deviation (SD). Values within a row with a symbol indicate statistically significant differences. ^†^ *p* < 0.05 versus colostrum. ^‡^ *p* < 0.05 versus transitional milk.

**Table 3:** Median phospholipids composition of HM expressed as mg/100 mL.

mg/100 mL	Total Population			Guangzhou			Beijing			Suzhou		
	Colostrum (0–5 Days) *n* = 113	Transitional (6–15 Days) *n* = 81	Mature (16 Days–8 Months) *n* = 345	Colostrum (0–5 Days) *n* = 38	Transitional (6–15 Days) *n* = 22	Mature (16 Days–8 Months) *n* = 120	Colostrum (0–5 Days) *n* = 45	Transitional (6–15 Days) *n* = 21	Mature (16 Days–8 Months) *n* = 113	Colostrum (0–5 Days) *n* = 30	Transitional (6–15 Days) *n* = 38	Mature (16 Days–8 Months) *n* = 112
PtdCho	12.0 ± 5.8	10.1 ± 5.5 ^†^	8.2 ± 5.0 ^†,‡^	12.5 ± 4.6 ^a^	11.3 ± 5.6 ^b^	8.6 ± 5.1 ^c^	10.9 ± 4.8 ^a^	8.3 ± 3.7 ^b^	7.6 ± 4.5 ^c^	12.6 ± 7.7 ^a^	11.9 ± 6.1 ^b^	8.5 ± 5.3 ^c^
CerPCho	9.1 ± 4.0	7.3 ± 4.1 ^†^	7.2 ± 4.0 ^†^	7.7 ± 1.6 ^a^	6.8 ± 2.7 ^b^	7.1 ± 4.0 ^c^	10.9 ± 4.9 ^d^	6.2 ± 3.8 ^b^	7.3 ± 3.9 ^c^	9.7 ± 3.1 ^a^	8.5 ± 4.7 ^e^	7.4 ± 4.2 ^c^
PtdEtn	8.5 ± 5.2	8.2 ± 5.3	6.4 ± 3.4 ^†,‡^	9.9 ± 2.6 ^a^	5.6 ± 3.7 ^b^	7.1 ± 3.9 ^c^	7.6 ± 3.1 ^d^	7.3 ± 2.4 ^b^	5.3 ± 2.6 ^e^	12.6 ± 7.4 ^f^	10.8 ± 5.8 ^g^	7.3 ± 3.2 ^c^
PtdIns	1.8 ± 0.7	1.8 ± 1.0	1.5 ± 0.7 ^†,‡^	1.8 ± 0.5 ^a^	1.2 ± 0.7 ^b^	1.5 ± 0.8 ^c^	1.6 ± 0.5 ^a^	1.5 ± 0.4 ^b^	1.2 ± 0.5 ^d^	2.3 ± 1.0 ^e^	2.4 ± 1.1 ^f^	1.7 ± 0.8 ^g^
PtdSer	1.5 ± 1.6	1.1 ± 0.8 ^†^	1.0 ± 1.0 ^†^	1.3 ± 0.4 ^a^	0.8 ± 0.4 ^b^	1.0 ± 0.6 ^c^	1.8 ± 2.3 ^d^	1.0 ± 1.4 ^b^	0.9 ± 1.2 ^c^	1.7 ± 0.5 ^a^	1.3 ± 0.5 ^e^	1.2 ± 1.4 ^f^
**Total PL (mg/100 mL)**	33.0 ± 13.2	28.5 ± 14.4 ^†^	24.2 ± 11.4 ^†,‡^	33.2 ± 8.1 ^a^	25.6 ± 11.1 ^b^	25.3 ± 12.5 ^c^	33.0 ± 11.2 ^a^	24.4 ± 8.1 ^b^	22.3 ± 9.9 ^d^	38.9 ± 18.8 ^e^	34.9 ± 16.6 ^f^	26.02 ± 11.3 ^c^

PL, phospholipids. * Values are presented as median ± standard deviation. Values within a row with a symbol indicate statistically significant differences. ^†^ *p* < 0.05 versus colostrum. ^‡^ *p* < 0.05 versus transitional milk. ^a,b,c,d,e,f,g^ *p* < 0.05 among cities.

**Table 4:** Average GD composition of HM expressed as mg/L.

	Total Population			Guangzhou			Beijing			Suzhou		
GD mg/L	Colostrum (0–5 Days) *n* = 113	Transitional (6–15 Days) *n* = 81	Mature (16 Days–8 Months) *n* = 345	Colostrum (0–5 Days) *n* = 38	Transitional (6–15 Days) *n* = 22	Mature (16 Days–8 Months) *n* = 120	Colostrum (0–5 Days) *n* = 45	Transitional (6–15 Days) *n* = 21	Mature (16 Days–8 Months) *n* = 113	Colostrum (0–5 Days) *n* = 30	Transitional (6–15 Days) *n* = 38	Mature (16 Days–8 Months) *n* = 112
**GM3**	3.8 ± 2.5	5.5 ± 3.2 ^†^	10.1 ± 4.6 ^†,‡^	4.0 ± 2.7 ^a^	7.7 ± 4.5 ^b^	10.5 ± 4.6 ^c^	3.7 ± 2.3 ^a^	3.3 ± 1.6 ^d^	9.0 ± 3.8 ^c^	4.0 ± 2.6 ^a^	5.4 ± 2.0 ^e^	10.8 ± 5.2 ^c^
**GD3**	4.1 ± 4.5	3.0 ± 3.4 ^†^	1.0 ± 1.7 ^†,‡^	2.8 ± 2.5 ^a^	3.0 ± 3.5 ^b^	1.0 ± 2.3 ^c^	2.2 ± 2.0 ^a^	3.0 ± 2.8 ^b^	0.7 ± 0.9 ^c^	8.6 ± 5.9 ^d^	2.9 ± 3.7 ^b^	1.1 ± 1.5 ^c^
**GM3 + GD3**	8.0 ± 5.3	8.5 ± 4.5 ^†^	11.0 ± 5.0 ^†,‡^	6.6 ± 3.2 ^a^	10.7 ± 4.7 ^b^	11.5 ± 5.1 ^c^	5.9 ± 2.7 ^a^	6.3 ± 3.4 ^d^	9.7 ± 4.0 ^c^	12.6 ± 7.0 ^e^	8.3 ± 4.3 ^f^	11.9 ± 5.6 ^c^
**GM3/GD3**	0.9	1.8 ^†^	10.1 ^†,‡^	1.4	2.6	10.4	1.7	1.1	12.5	0.5	1.8	9.4

* Values are presented as average ± standard deviation. Values within a row with a symbol indicate statistically significant differences. ^†^ *p* < 0.05 versus colostrum. ^‡^ *p* < 0.05 versus transitional milk. ^a,b,c,d,e,f^ *p* < 0.05 among cities.

## Abbreviations

FA (FA), phospholipids (PL), gangliosides (GD), gas chromatography (GC), evaporative light scattering detector (ELSD), with time of flight (TOF), triacylglycerols (TAG), saturated FA (SFA), mono-unsaturated (MUFA), polyunsaturated (PUFA), long chain polyunsaturated FA (LCPUFA), linoleic (LA), linolenic acids (ALA), arachidonic (ARA), docosahexaenoic (DHA), phosphatydylinositol (PtdIns), phosphatydylethanolamine (PtdEtn), phosphatydylserine (PtdSer), phosphatidylcholine (PtdCho), sphingomyelin (CerPCho), methyl esters of FA (FAMEs).
